# PRMT7 Inhibits the Proliferation and Migration of Gastric Cancer Cells by Suppressing the PI3K/AKT Pathway via PTEN

**DOI:** 10.7150/jca.88102

**Published:** 2023-09-04

**Authors:** Xuemei Wang, Wenfang Xu, Chongyang Zhu, Yu Cheng, Jiemin Qi

**Affiliations:** Department of Pathology, Chengde Medical University, Chengde, Hebei 067000, People's Republic of China.

**Keywords:** Gastric cancer, PRMT7, PTEN, PI3K/AKT, Arginine methylation

## Abstract

Protein arginine methyltransferase 7 (PRMT7) plays a crucial role in tumor occurrence and development; however, its expression pattern, biological function, and specific mechanism in gastric cancer (GC) remain poorly defined. The present study aimed to investigate the role of PRMT7 during GC carcinogenesis and its underlying mechanism. We found that PRMT7 is expressed at low levels in GC tissues, and this low expression is associated with tumor size, differentiation degree, lymph node metastasis, and TNM stage. Functionally, PRMT7 inhibits GC cell proliferation and migration. Mechanistically, PRMT7 induces PTEN expression and suppresses the downstream PI3K/AKT signaling cascade. Finally, we confirmed that PRMT7 interacts with PTEN protein and promotes PTEN arginine methylation. Taken together, our findings suggest that PRMT7 can inhibit PI3K/AKT signaling pathway activation by regulating PTEN, thereby inhibiting GC cell proliferation and migration. PRMT7 may be a promising therapeutic target for the prevention of GC.

## Introduction

Gastric cancer (GC) is one of the most common gastrointestinal malignancies worldwide. According to the latest data from the International Agency for Research on Cancer (IARC) of the World Health Organization (WHO), the number of GC cases diagnosed every year is as high as 1,089 million, and the number of deaths is nearly 769,000 1. Owing to the low diagnostic rate and the lack of obvious symptoms of early GC, most patients are already at middle or advanced stages when initially diagnosed. Therefore, despite the development of surgical techniques and the implementation of radiotherapy, chemotherapy, and neoadjuvant therapy, the 5-year survival rate of patients with advanced GC after surgery is <25% 2,3. Therefore, additional potential biomarker screening for early GC diagnosis, finding potential therapeutic targets, and exploring the biological behavior and regulatory mechanism of GC are useful goals.

Protein arginine methylation is a common post-translational modification (PTM) process catalyzed by a group of enzymes, namely protein arginine methyltransferases (PRMTs) 4. PRMTs regulate various cellular functions, such as signal transduction, protein-protein interaction, transcription regulation, and mRNA splicing, that are intimately associated with progression to multiple diseases, including cancers 5,6. PRMT7 is a signal type III PRMT that solely catalyzes arginine monomethylarginine (MMA) formation on histones and non-histone proteins, mediating epigenetic changes, such as DNA methylation, mRNA cleavage, and protein PTM 7-9. Aberrant PRMT7 expression has been reported in human malignancies and plays vital roles in the progression of cancer. PRMT7 was identified as an oncogene in breast, lung and renal cell carcinoma cancers and involved in the proliferation, invasion and metastasis of cancer cells by mediating the EMT or cancer-related signaling pathways 9-11. However, PRMT7 was recently shown to restrain melanoma growth by regulating immune checkpoint 12. It can thus be seen that the function of PRMT7 in malignant tumor are divers and the underlying mechanisms is complicated as well. However, the expression pattern, biological function, and underlying mechanisms of PRMT7 in GC remain unclear.

PTEN, whose role as a tumor suppressor has been extensively studied, is a prominent negative modulator of the PI3K/AKT signaling pathway. Under physiological conditions, receptor tyrosine kinases and G protein-coupled receptors activate PI3K, which in turn catalyzes the phosphorylation of the lipid substrate PIP2 to produce PIP3. PIP3 accumulation is tightly controlled by PTEN, which directly antagonizes the activation of the PI3K/AKT signaling pathway and regulates biological cellular functions. Thus, PTEN has been conclusively shown to be involved in tumor occurrence and progression 13-15. The PI3K protein family contains three lipid kinases, of which class I kinases are particularly important for cell proliferation and tumor formation and consist of the catalytic and regulatory subunits p110 and p85 16. AKT can be activated by Ser473 phosphorylation of p-AKT, which participates in a variety of biological processes, such as proliferation, migration, and apoptosis 17,18. PTEN can also be regulated by extensive PTMs, including acetylation, oxidation, phosphorylation, SUMOylation, and ubiquitination 19. However, modification of PTEN by arginine methylation is infrequently reported. A recent study reported that PTEN can be arginine-dimethylated by PRMT6 20; however, whether PTEN is a substrate of PRMT7 is unknown.

This study aimed to investigate the possible biological functions and underlying molecular mechanisms of PRMT7 in GC. We demonstrated that PRMT7 inhibited the proliferation and migration of GC cells by suppressing the PI3K/AKT pathway via PTEN arginine methylation.

## Materials and Methods

### Patients and tissues

Paraffin samples of GC were obtained from the Affiliated Hospital of Chengde Medical College and Wuhan Shuangxuan Biotechnology Co., Ltd. (IWT-N-70G72, IWT-N-96G43), including 152 GC tissue samples and 69 adjacent gastric mucosa tissue samples (control samples). In addition, 30 fresh GC tissues and paired control mucosa tissues were collected and stored at -80℃ for 5 min in vitro for subsequent western blot experiments. Patient inclusion criteria were (1) All GC patients were newly diagnosed in pathological examinations, (2) the clinical and pathological data of all cases were complete, (3) all patients underwent operative treatment and none of the them had received any preoperative radiotherapy or chemotherapy. All patients provided informed consent, and all experiments were approved by the Research Ethics Committee of Chengde Medical College (No. 2022002).

### Immunohistochemical (IHC) analysis

All tissue samples were stained immunohistochemically using the UltraSensitiveTM SP (Mouse/Rabbit) kit (KIT-9710, MXB Biotechnologies, Fuzhou, China), and tissue sections were heated at 60°C for 2 h, dewaxed in xylol, and subjected to gradient alcohol hydration. Following sodium citrate antigen repair for 3 min, the antigen was extracted at high temperature and pressure following the instruction manual. Sections were incubated with rabbit anti-Homo sapiens (Human) PRMT7 polyclonal antibody (CSB-PA885738LA01HU, CUSABIO, Wuhan, China) at 1:100 dilution overnight at 4°C followed by a goat anti-rabbit/mouse secondary antibody. DAB and hematoxylin were used for nuclear staining. Finally, the sections underwent dehydration in gradient alcohol, cleared in xylene, and sealed with neutral gum.

The immunohistochemical results of PRMT7 were evaluated in a double-blind manner. The IHC score equaled to the product of the staining intensity and the percentage of positive cells. The staining intensity scores were as follows: 0, no staining; 1, weak staining; 2, moderate staining; and 3, strong staining. The percentage of positive cells was 1:0-25%, 2:26-50%, 3:51-70%, and 4:71-100% 21. The two scores were multiplied to obtain a score that ranged from 0 to 12. Specimens with a total score ≤6 were considered to show low PRMT7 expression, and those with scores >6 were considered to show high PRMT7 expression.

### Cell culture, transfection, and treatment

The human gastric carcinoma-derived cell lines AGS, MGC-803, BGC-823, and SGC-7901, and the immortalized gastric epithelial cell line, GES-1, were obtained from the Cell Bank of the China Academy of Sciences (Shanghai, China). AGS cells were cultured in DMEM/F12 medium, and GES-1, MGC-803, BGC-823, and SGC-7901 cells were cultivated in RPMI 1640 medium. Fetal bovine serum (FBS; Biological Industries, 04-001-1A, Beth Haemek, Israel) (10%) was added to all media, and the cells were incubated at 37°C under 5% CO_2_ conditions.

Two PRMT7 small interfering RNAs (siRNAs) (RiboBio, Guangzhou, China) or a PRMT7 overexpression plasmid (Sino Biological, HG20524-CH, Beijing, China) were transfected into the gastric cell lines MGC-803 and AGS using Lipofectamine 3000 reagent (Invitrogen, Carlsbad, CA, USA), according to the manufacturer's protocol. The targeting sequence of siPRMT7#1 was 5'-GTCACAGAGTTGTTTGACA-3' and that of siPRMT7#2 was 5'-GCTACATGACAAAGACAGA-3'. The targeting sequence of siPTEN was 5'-TCTTCAAAAGGATATTGTGCA-3' 22. The DNA fragment of PRMT7 was cloned into the PCMV3 expression vector containing the FLAG sequence 5'-GGCAACTAGAAGGCACAGTCGAGG-3'. For PI3K/AKT signaling pathway inhibitor treatment, 50 μM LY294002 (MedChemExpress, Monmouth Junction, NJ, USA), which blocks the PI3K/AKT pathway, was added to the cells 36 h after PRMT7 overexpression plasmid transfection, and the same amount of DMSO was added to the control cells.

### Cell counting kit-8 (CCK-8) and colony formation assays

The cells were counted and inoculated in a 96-well plate at a density of 3000 cells/well, and 100 μL of medium containing 10% FBS was added to each well at 24 h after transfection. The experimental and control groups were each set up with five replicate wells. Cells were incubated with 10% CCK-8 solution (APExBIO, K1018, Houston, TX, USA) at 100 μL/well for 1 h. Absorbance at 450 nm was measured once daily for 5 consecutive days. For colony formation assays, the cells were counted and inoculated in a 6-well plate at a density of 1000 cells/well, and 3 mL of 10% FBS was added to each well 24 h post-transfection. After 7-14 days of incubation, cells were fixed with cold methyl alcohol for 30 min and stained with 0.5% crystal violet for 30 min. Subsequently, the ImageJ software was used to count the colonies.

### Transwell migration assay

After 24 h of transfection, GC cells were counted and inoculated into 24-well Transwell chambers with 8 μm pores. Approximately 60,000 MGC-803 cells or 30,000 AGS cells were added to the upper chamber in 200 μL culture medium with 2% FBS, and culture medium with 20% FBS (600 μL) was added to the lower chamber. After culturing for 18-20 h at 37°C and under 5% CO_2_ conditions, cells were fixed with cold methyl alcohol for 30 min and stained with 0.5% crystal violet for 30 min. A cotton swab was used to gently remove non-migrant cells from the upper side of the membrane, and the migrated cells were observed and photographed under a high-power microscope.

### Western blotting analysis

RIPA lysis buffer (R0020, Solarbio, Beijing, China) with inhibitors for protein protease and phosphatase was used to extract total tissue and cellular proteins on ice, and the protein was quantified using the BCA method. Subsequently, 30 μg/lane total protein was run by sodium dodecyl-sulfate polyacrylamide gel electrophoresis (SDS-PAGE) (10% agarose gel), electro-transferred onto polyvinylidene difluoride (PVDF) membranes (0.45 μm), and blocked with 5% skim milk or bovine serum albumin at 37℃ for 2 h. The PVDF membranes were then incubated with the indicated primary antibodies (Table 1) overnight at 4°C. The next day, the membranes were washed thrice with TBST and incubated with goat anti-rabbit/mouse IgG conjugated with horseradish peroxidase at 37℃ for 1 h. Immunoreactive signals were detected using ECL plus on a ChemiDoc MP imaging system (Bio-Rad, Hercules, CA, USA).

### Quantitative real-time PCR (RT-qPCR)

Total RNA was extracted from cells using TRIzol reagent (BS259A, Biosharp, Beijing, China) and reverse-transcribed to cDNA using a FastQuant RT kit (TIANGEN Biotech, Beijing, China) according to the manufacturer's protocol. RT-qPCR was performed using a Bio-Rad CFX96 Fast Real-Time PCR Detection System. The PCR amplification cycling conditions were as follows: 15 min at 95°C, denaturing for 10 s at 95°C, and annealing at 60°C for 32 s for 40 cycles. The 2-ΔΔCq method was used to calculate the relative mRNA expression, and GAPDH was selected as an internal control.

PCR primers were synthesized by Sangon Biotech (Shanghai, China), and the sequences are as follows: PRMT7 forward: 5'-CTGGAGGAGGATGAACACTATG-3', and reverse: 5'-CCCGGATACCTTGGTAGTATTT-3'; PTEN forward: 5'-TGGATTCGACTTAGACTTGACCT-3', and reverse: 5'-GGTGGGTTATGGTCTTCAAAAGG-3'; GAPDH forward: 5'-GCACCGTCAAGGCTGAGAAC-3', and reverse: 5'-TGGTGAACGCCAGTGGA-3'.

### Co-immunoprecipitation

Briefly, the cells were gently scraped using a cell scraper, pelleted, and lysed for 30 min on ice with an appropriate amount of lysate. The supernatant was blocked with 60 μL Protein A+G agarose beads (Beyotime Biosciences) for 2 h at 4°C and then centrifuged at 2000 rpm for 5 min to remove the beads. The supernatant was divided, and anti-PTEN antibody (#22034-1-AP, Proteintech), anti-His antibody (#66005-1-Ig, Proteintech), or control anti-mouse/rabbit IgG was added and incubated at 4°C overnight with rotation. The next day, 60 μL Protein A+G agarose beads were used to capture the immunocomplexes, and coprecipitating proteins were detected by western blot experiments.

### In vitro methylation assays

In brief, PRMT7 was knocked down or overexpressed in AGS cells, which were harvested and lysed 48 h after transfection and subjected to immunoprecipitation with anti-PTEN antibody overnight at 4°C. The following day, the immune complexes were pulled down using Protein A + G agarose beads at 4°C for 6 h and washed thrice in IP lysis buffer. Immune precipitates were assayed by western blotting with an anti-MMA antibody (1:1000, #8015S, CST).

### Statistical analysis

The experimental data were statistically analyzed using SPSS software (version 26.0; SPSS Inc.) and GraphPad Prism software (version 9.0; GraphPad Software, Inc.) Correlations between PRMT7 protein expression and clinicopathological parameters were determined using the chi-square test. Unless otherwise stated, quantitative data are expressed as the mean ± standard error and compared using Student's t-test. Significance was set as follows: *P < 0.05, **P < 0.01, and ***P < 0.001. Data represent ≥3 independent replicates.

## Results

### PRMT7 is downregulated in GC

**Figure 1:**
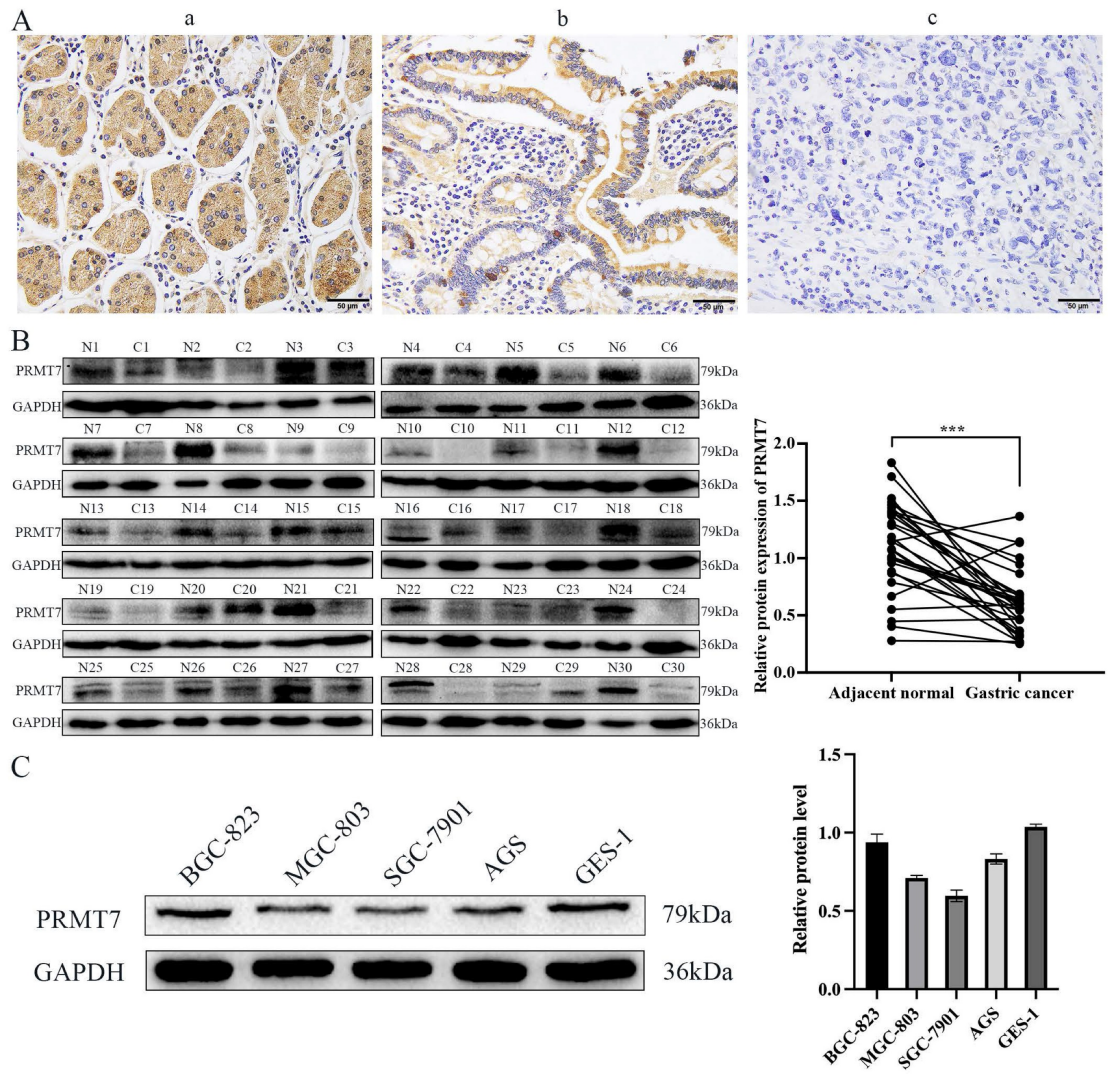
PRMT7 is expressed at low levels in gastric cancer tissues. **A** Immunohistochemical analyses were performed to observe PRMT7 expression in gastric cancer: a) normal gastric tissue; b) high-medium differentiated gastric cancer tissue; c) poorly differentiated gastric cancer tissue. B Western blot analysis of PRMT7 protein expression levels in 30 paired gastric cancer tissues and adjacent normal mucosal tissues. N: normal mucosa adjacent to cancer; T: tumor tissue. **C** PRMT7 expression in the GES-1 cell line and various gastric cancer cell lines. ***P < 0.001.

To investigate the expression pattern of PRMT7 in GC, 152 cases of GC and 69 cases of adjacent control mucosa were detected using the immunohistochemical SP method. Our data showed that PRMT7 expression was significantly lower in GC tissues (positivity rate: 34.8%) than in control tissues (positivity rate: 62.3%), indicating that PRMT7 expression was low in GC tissues (Table 2, Fig. 1A). As shown in Table 3, PRMT7 expression was significantly negatively correlated with GC size, differentiation degree, lymph node metastasis, invasion depth, and TNM stage but not with sex and age (Table 3). PRMT7 protein expression in 30 paired fresh-frozen tumor and noncancerous tissues was detected through western blotting. PRMT7 expression was significantly lower in 25 of the 30 GC tissues than in the paired adjacent mucosa (Fig. 1B). PRMT7 expression in GC cell lines (AGS, MGC-803, BGC-823, and SGC-7901) and the control gastric epithelial cell line GES-1 was determined using western blotting. PRMT7 levels were reduced in GC cell lines compared to those in GES-1 cells (Fig. 1C). These results suggest that the abnormally low expression of PRMT7 in GC tissues and cells may be closely related to GC occurrence and development.

### PRMT7 inhibited GC cell proliferation and migration

**Figure 2:**
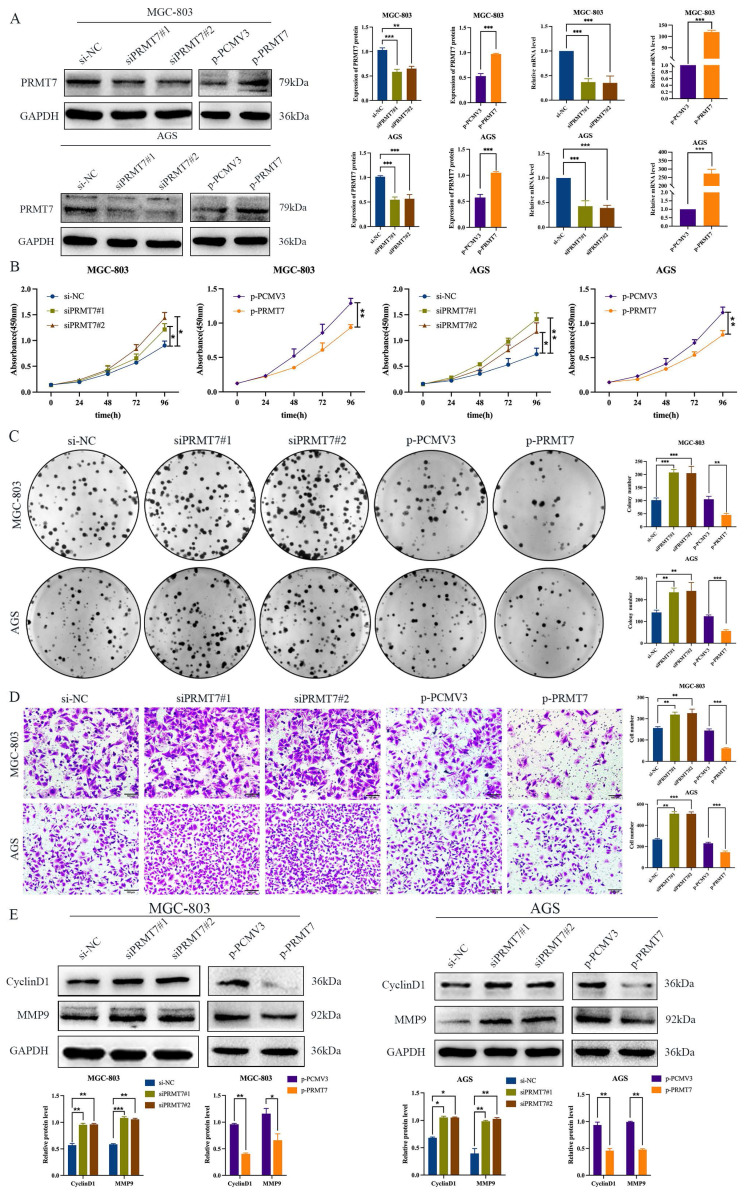
PRMT7 inhibited gastric cancer cell proliferation and migration. **A** The knockdown and overexpression efficiency of the PRMT7 was detected by western blot and RT-qPCR. **B** CCK-8 assays were performed to compare experimental groups with the si-NC group, and PRMT7 downregulation promoted cell growth, while PRMT7 overexpression did not. **C** Colony formation experiments were performed and the number of colonies in the experimental group was compared to that in the si-NC group, and PRMT7 downregulation promoted cell proliferation, while PRMT7 overexpression did not. **D** Transwell experiments were performed to determine the migration of experimental cells compared to that of the si-NC group cells, and PRMT7 downregulation promoted cell migration, while PRMT7 overexpression did not. **E** Effects of low/high PRMT7 expression on the expression of proliferation- and migration-related proteins were analyzed by western blot and quantified based on relative grey values. *P < 0.05, **P < 0.01, and ***P < 0.001.

To further explore the potential biological role of PRMT7 in GC cells, we selected MGC-803 and AGS cells with moderate PRMT7 expression for transfection with siRNA and plasmid DNA based on the results presented in Fig. 1C. The knockdown or overexpression efficiency of PRMT7 was assessed using RT-qPCR and western blotting. The mRNA and protein levels of PRMT7 were reduced in the PRT7-siRNA-transfected group, but they were overexpressed in the PCMV3-PRMT7 vector-transfected group (Fig. 2A). CCK-8 assay results showed that PRMT7 knockdown significantly promoted the viability of MGC-803 and AGS cells compared to that of the control group. In contrast, PRMT7 overexpression significantly inhibited the viability of these cells (Fig. 2B). We also found that PRMT7 knockdown significantly increased the number of colonies formed in MGC-803 and AGS cells compared to that in the control cells, while PRMT7 overexpression significantly inhibited this effect (Fig. 2C). Similarly, the Transwell assay results showed that PRMT7 inhibited the migration ability of GC cells (Fig. 2D). In addition, PRMT7 downregulated the proliferation-related protein CyclinD1 and migration-related protein MMP9 expression at the protein level (Fig. 2E). These results indicated that PRMT7 could inhibit the proliferation and migration of GC cells in vitro.

### PRMT7 regulates PTEN and its downstream PI3K/AKT signaling pathway

**Figure 3:**
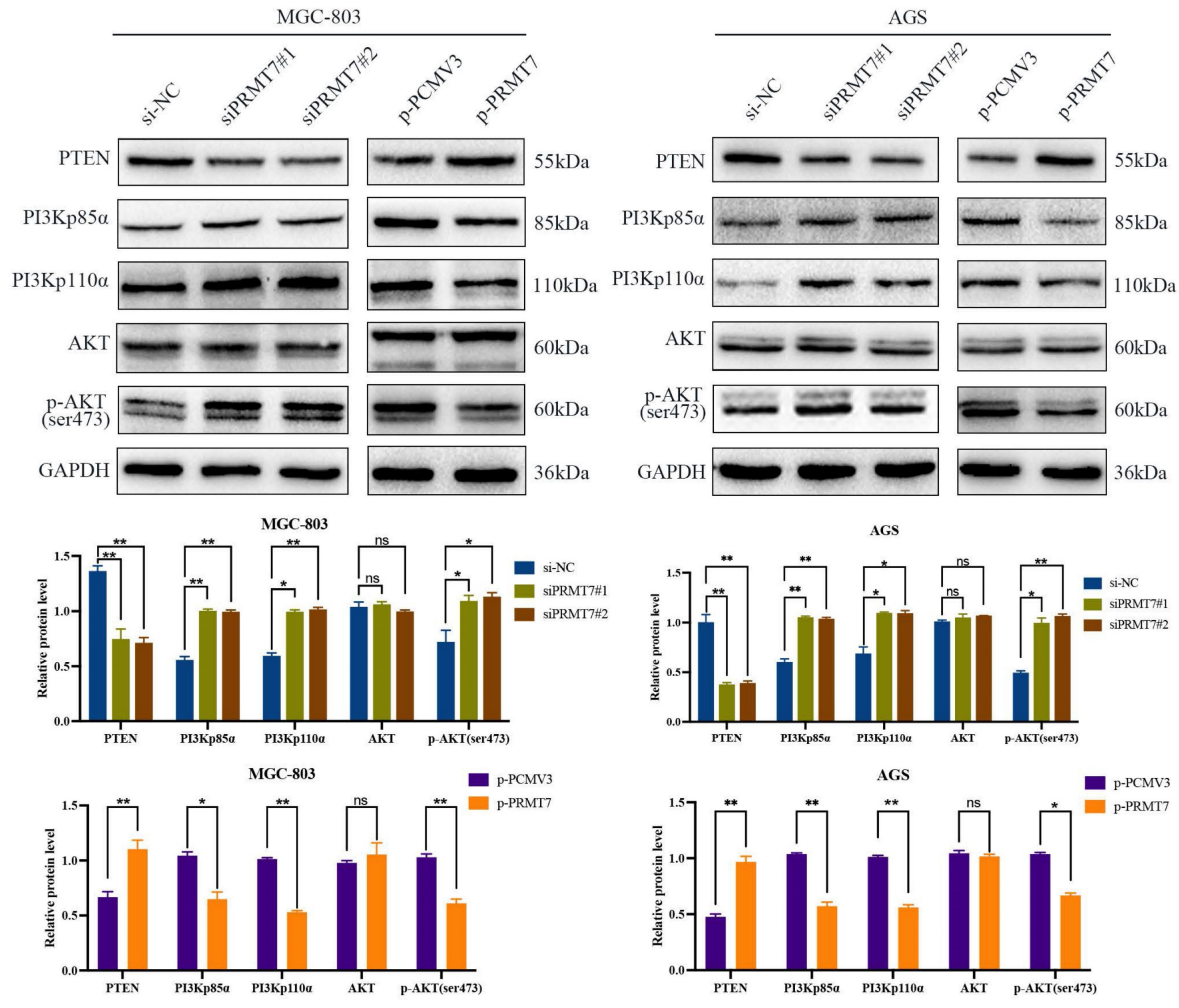
Effect of PRMT7 expression on PTEN and downstream PI3K/AKT signaling pathway. Western blot was used to detect the effect of low/high PRMT7 expression on the expression of PTEN and related proteins in the downstream PI3K/AKT signaling pathway. *P < 0.05, **P < 0.01, ***P < 0.001 and ns: no significance.

The PI3K/AKT pathway plays a crucial role in GC cell growth and survival and is negatively regulated by its upstream regulator PTEN. We postulated that PRMT7 may be involved in the regulation of PTEN and its downstream PI3K/AKT signaling pathway. Western blotting results revealed that PRMT7 depletion downregulated the expression level of PTEN, while the expression levels of PI3Kp110α, PI3Kp85α, and p-AKT/AKT were increased. In contrast, PRMT7 overexpression showed the opposite trend (Fig. 3). These data suggest that PRMT7 may regulate the PTEN and PI3K/AKT pathways in GC.

### PRMT7 executes tumor suppressor function via PTEN and its downstream PI3K/AKT signaling pathway

**Figure 4:**
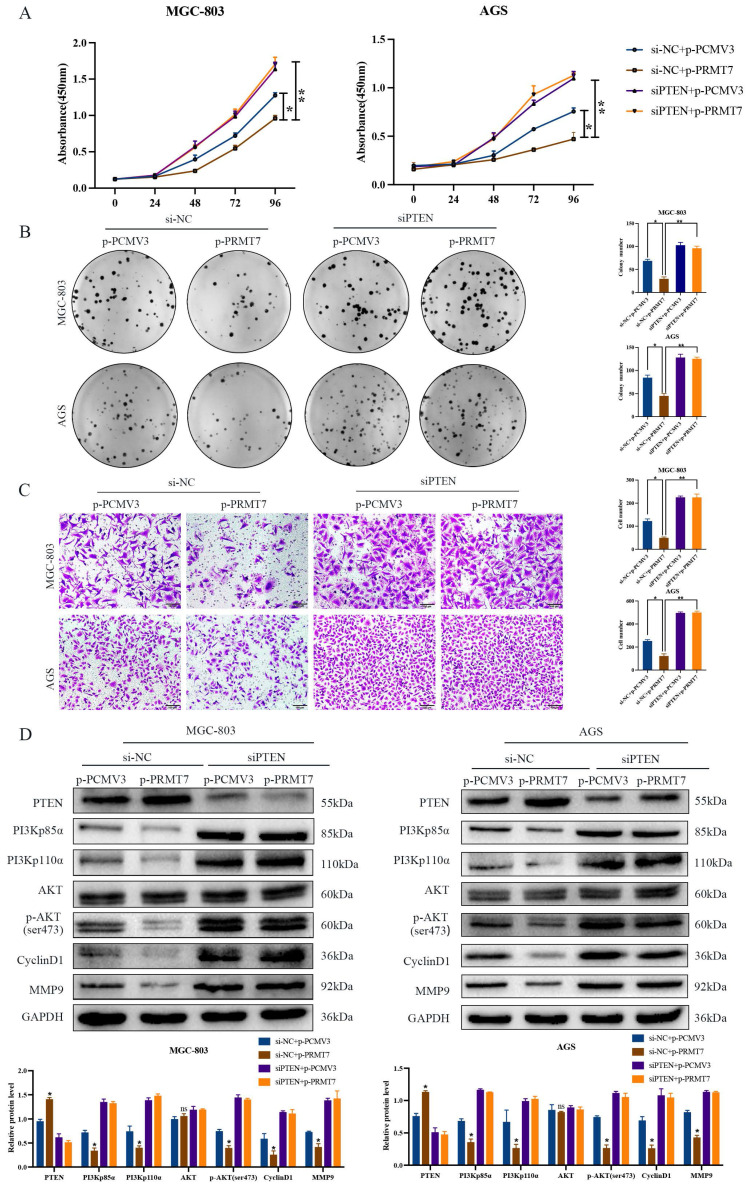
PRMT7 plays a tumor suppressor role dependent on PTEN. **A** Effect of PRMT7 overexpression on the viability of PTEN-deficient gastric cancer cells. **B** Effect of PRMT7 overexpression on the proliferation of PTEN-deficient gastric cancer cells. **C** Effect of PRMT7 overexpression on the migration of PTEN-deficient gastric cancer cells. **D** Knockdown of PTEN reversed the effect of PRMT7 upregulation on the expression of PTEN and its downstream signaling pathways and target proteins. *P < 0.05, **P < 0.01, ***P < 0.001 and ns: no significance.

To determine whether PTEN is required for PRMT7's suppressive effect, we designed a rescue experiment by inhibiting PTEN expression in PRMT7-overexpressing cells. As expected, PTEN depletion reversed the inhibitory effect of PRMT7 overexpression on the proliferation and migration of MGC-803 and AGS cells (Fig. 4A-C). In addition, knockdown of PTEN restored the effect of PRMT7 upregulation on the PTEN and PI3K/AKT signaling pathways and target proteins (Fig. 4D).

**Figure 5:**
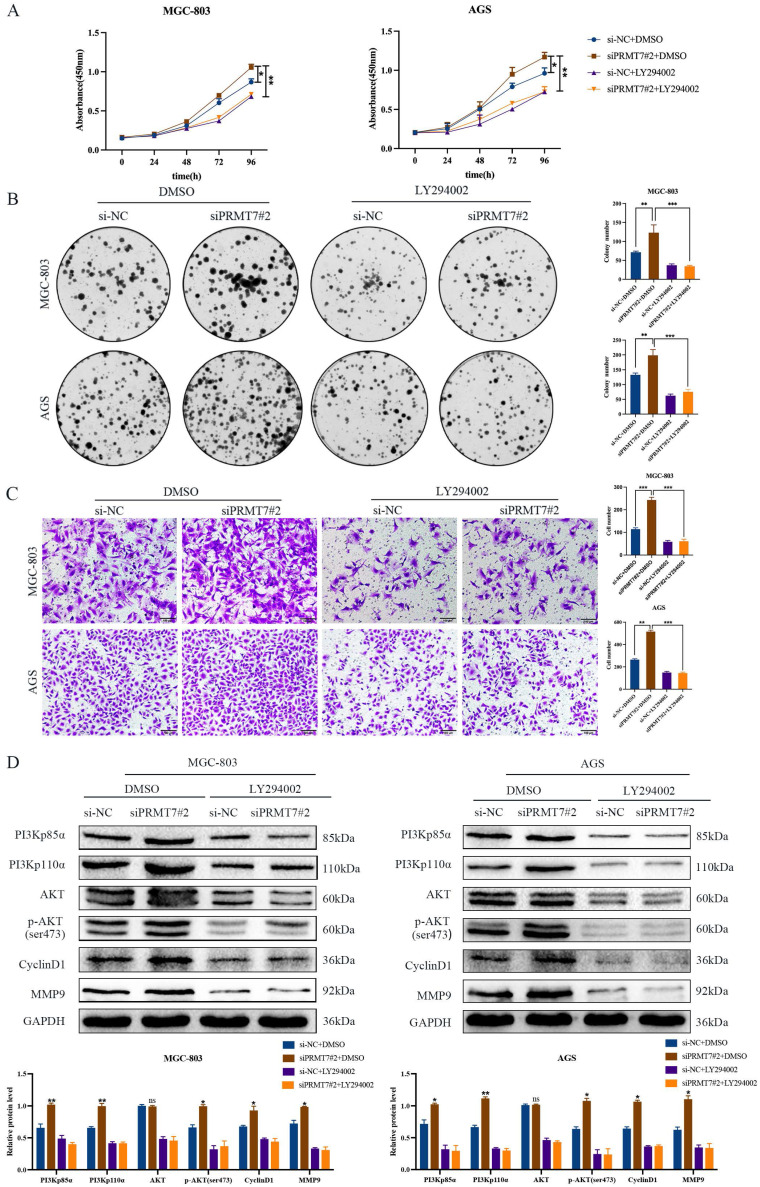
PRMT7 affects gastric cancer cell proliferation and migration via the PI3K/AKT signaling pathway. **A** CCK-8 assay was used to detect the effect of LY294002 on the viability of gastric cancer cells. **B** Colony formation assay was used to detect the effect of LY294002 on the proliferation of gastric cancer cells. **C** Transwell assay was used to detect the effect of LY294002 on the migration ability of gastric cancer cells. **D** LY294002 reversed the effect of PRMT7 knockdown on PI3K/AKT signaling. *P < 0.05, **P < 0.01, ***P < 0.001 and ns: no significance.

To further confirm whether PRMT7 acts via the PI3K/AKT signaling pathway, we treated siPRMT7#2-transfected cells with a PI3K/AKT inhibitor (LY294002) and then observed the proliferation, migration, and protein expression changes in GC cells. Our results indicated that LY294002 significantly inhibited the proliferation and migration of PRMT7-overexpressing cells (Fig. 5A-C). In addition, western blotting showed that LY294002 reversed the effect of PRMT7 overexpression on the protein expression of PI3Kp110α, PI3Kp85α, p-AKT/AKT, CyclinD1, and MMP9 (Fig. 5D). Our results conclusively show that PRMT7 inhibited the proliferation and migration of GC cells by regulating the PI3K/AKT signaling pathway in a PTEN-dependent manner.

### PRMT7 interacts with PTEN and mediates PTEN arginine methylation in GC cells

**Figure 6:**
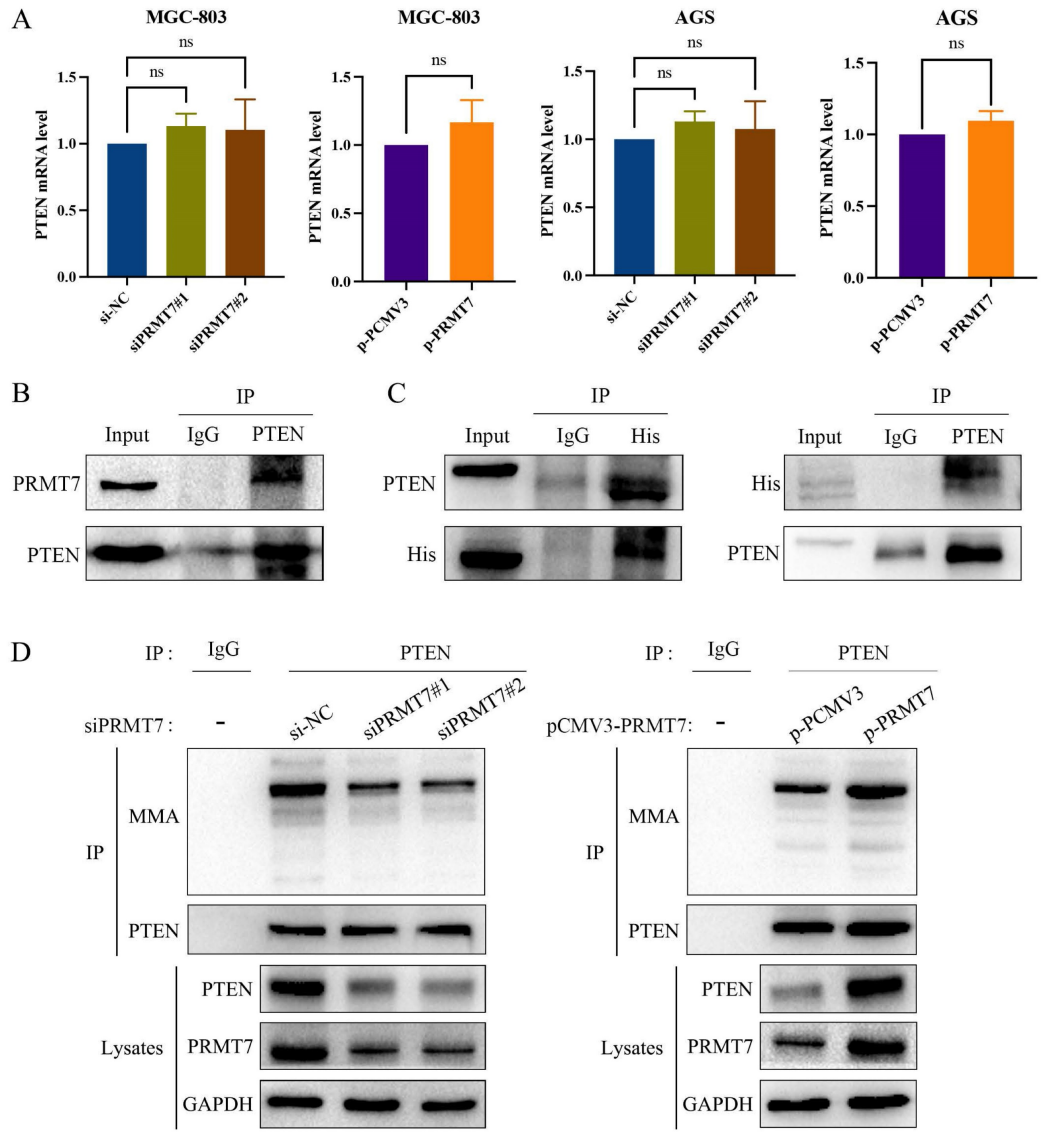
PRMT7 interacts with PTEN and promotes PTEN methylation. **A** RT-qPCR was used to detect the expression of PTEN mRNA in the transfected cells. **B** Endogenous PTEN and PRMT7 immunoprecipitation occurred in AGS cells. **C** Exogenous PTEN and PRMT7 co-immunoprecipitated in AGS cells. **D** AGS cells transfected with PRMT7 siRNA and overexpression plasmid were subjected to co-immunoprecipitation with PTEN antibody, followed by detection of PTEN methylation level with MMA antibody. *P < 0.05, **P < 0.01, ***P < 0.001 and ns: no significance.

PRMT7 knockdown suppressed PTEN expression, and PRMT7 overexpression upregulated PTEN expression at the protein level. However, as shown in Fig. 6A, PTEN mRNA levels were not significantly altered by the changes in PRMT7 expression. This finding suggests that PRMT7 may modulate PTEN expression at the post-transcriptional level. PRMT7 itself is an arginine methyltransferase and can exert methylation independently, so we hypothesized that PRMT7 may bind to PTEN and participate in the methylation modification of the PTEN protein. To test this hypothesis, we performed endogenous immunoprecipitation with an anti-PTEN antibody in AGS cells to determine whether endogenous PRMT7 binds endogenous PTEN. As per our hypothesis, PRMT7 was detected in the PTEN precipitated immunocomplex (Fig. 6B). Additionally, we transfected the exogenous His-tagged PRMT7 plasmid into AGS cells, followed by immunoprecipitation of the cell lysates with anti-His or anti-PTEN antibodies. The results showed that exogenous PRMT7 co-immunoprecipitated with PTEN (Fig. 6C). To further elucidate whether PRMT7 could regulate the methylation level of PTEN, we performed a co-immunoprecipitation assay with PTEN antibody, followed by a western blot assay with MMA antibody. The results showed that the PTEN methylation level in the siPRMT7 group was significantly reduced compared to that in the control group but increased in the p-PRMT7 group (Fig. 6D). These results indicated that PRMT7 interacts with PTEN and mediates PTEN arginine methylation.

## Discussion

PRMT7 was downregulated in GC tissues, and its expression correlated with tumor size, differentiation degree, lymph node metastasis, TNM stage, and invasion depth. We also found that PRMT7 inhibits the proliferation and migration of GC cells in vitro. These results suggest that PRMT7 inhibits GC development and progression. PRMT7 is a key factor in the diagnosis of cell proliferation and distant metastasis in breast 23, liver 24, and lung 25 cancers, and the unlimited proliferation and distant metastasis of cancer cells are important causes of recurrence and death in patients 26. However, the specific biological function of PRMT7 in GC has not yet been reported. Therefore, it is imperative to explore the specific regulatory mechanisms of PRMT7 in GC.

The PI3K/AKT signaling pathway is a key regulator of the occurrence and development of malignant tumors, including GC, and its oncogenic effect on the tumorigenic phenotype of cancer cells has been confirmed 27-29. As the key downstream targets of the PI3K/AKT pathway, the cell cycle regulators Cyclin D1 and matrix metalloproteinase MMP9 play a major role in the regulation of cell proliferation and migration 30-32. PTEN mainly inactivates the phosphatidylinositol substrates required for PI3K signaling and loses the function of the downward transmission of PI3K molecular signals, which in turn leads to the inhibition of AKT phosphorylation and then regulates the biological function of cancer cells 15,33. In our study, we found that PRMT7 suppressed the activation of the PI3K/AKT signaling pathway and the downstream target proteins cyclinD1 and MMP9 by regulating PTEN and subsequently downregulating the expression levels of PI3Kp110α and PI3Kp85α to reduce the phosphorylation level of AKT. It is important to mention that siPTEN and PI3K inhibitors can partially restore the phenotypes induced by PRMT7 upregulation in GC cells. These results suggest that PRMT7 inhibits the proliferation and migration of GC cells by regulating PTEN and the downstream PI3K/AKT pathway.

PRMT7 is a key component of methylation, which is crucial for histone and non-histone methylation modification regulation and catalyzes MMA production 9. PTEN is regulated by the protein arginine methyltransferase PRMT5 34. Moreover, PTEN can be arginine methylated at the R159 site by PRMT6 20. PRMT5 and PRMT6 are members of the PRMT family and are highly homologous to PRMT7 35. Our co-immunoprecipitation and methylation experiments revealed that PRMT7 interacts with PTEN and promotes its MMA methylation. Nevertheless, it is not yet possible to identify which arginine site of PTEN is methylated by PRMT7. Therefore, further studies are warranted.

In conclusion, our results suggest that PRMT7 is involved in the tumorigenesis and progression of GC and inhibits the proliferation and migration of GC cells by binding to PTEN to promote its methylation modification, thereby regulating the downstream PI3K/AKT signaling pathway. PRMT7 may serve as a new biomarker and target for precise treatment of GC.

Taken together, our results reveal that PRMT7 regulates the PI3K/AKT signaling pathway by modifying PTEN via methylation to inhibit the proliferation and migration of GC cells, revealing the clinical significance and biological function of PRMT7 in GC. PRMT7 may represent an important potential target for GC therapeutics and may provide a new strategy for GC prevention, diagnosis, and treatment.

## Figures and Tables

**Table 1:** The antibodies used for Western blotting

Antibody Name	Source	Catalog Number	Host	Dilution
PRMT7	ABclonal	A17164	Rabbit	1:1000
GAPDH	Proteintech	60004-1-Ig	Mouse	1:10000
CyclinD1	Proteintech	60186-1-Ig	Mouse	1:2000
MMP9	ABclonal	A0289	Rabbit	1:1000
PI3Kp110α	Proteintech	67071-1-Ig	Mouse	1:2000
PI3Kp85α	Proteintech	60225-1-Ig	Mouse	1:2000
AKT	ABclonal	A18120	Rabbit	1:1000
p-AKT (ser473)	Affinity	AF0016	Rabbit	1:1000
PTEN	Proteintech	22034-1-AP	Rabbit	1:1000
MMA	CST	8015S	Rabbit	1:1000

**Table 2:** PRMT7 expression in para-carcinoma and gastric cancer tissues

Group	n	PRMT7		χ^2^	*P*
		High	Low		
Para-carcinoma	69	43	26	14.555	< 0.001
Gastric carcinoma	152	53	99		

**Table 3:** Association of PRMT7 expression with clinicopathological characteristics of the GC

Group	n	PRMT7		χ^2^	*P*
		High	Low		
Gender					
Male	117	40	77	0.104	0.748
Female	35	13	22		
Age					
≤60	80	25	55	0.974	0.324
>60	72	28	44		
Differentiation					
high-moderately	54	25	29	4.816	0.028
poorly	98	28	70		
Lymph node metastasis					
Yes	108	30	78	8.260	0.004
No	44	23	21		
TNM stage					
I+II	61	33	28	16.591	< 0.001
III+IV	91	20	71		
Depth of invasion					
≤muscle layer	58	26	32	4.096	0.043
>muscle layer	94	27	67		
Tumor size					
≤3cm	91	38	53	4.740	0.029
>3cm	61	15	46		
